# ICTV Virus Taxonomy Profile: *Arenaviridae* 2023

**DOI:** 10.1099/jgv.0.001891

**Published:** 2023-09-13

**Authors:** Sheli R. Radoshitzky, Michael J. Buchmeier, Rémi N. Charrel, Jean-Paul J. Gonzalez, Stephan Günther, Jussi Hepojoki, Jens H. Kuhn, Igor S. Lukashevich, Víctor Romanowski, Maria S. Salvato, Manuela Sironi, Mark D. Stenglein, Juan Carlos de la Torre

**Affiliations:** ^1^​ Center for Drug Evaluation and Research, Food and Drug Administration, Silver Spring, MD 20903, USA; ^2^​ University of California, Irvine, CA 92697, USA; ^3^​ Unité des Virus Emergents, Marseille CEDEX 05, France; ^4^​ Georgetown University, Washington, DC, USA; ^5^​ Bernhard Nocht Institute for Tropical Medicine, 20359 Hamburg, Germany; ^6^​ University of Helsinki, 00014 Helsinki, Finland; ^7^​ University of Zurich, Zurich, Switzerland; ^8^​ Integrated Research Facility at Fort Detrick, Fort Detrick, Frederick, MD 21702, USA; ^9^​ University of Louisville, Louisville, KY 40202, USA; ^10^​ Universidad Nacional de La Plata—Consejo Nacional de Investigaciones Científicas y Técnicas, 49 y 115 s/n La Plata, Argentina; ^11^​ University of Maryland, College Park, MD 20742, USA; ^12^​ Scientific Institute IRCCS “E. Medea”, 23842 Bosisio Parini, Italy; ^13^​ Colorado State University, Fort Collins, CO 80523, USA; ^14^​ The Scripps Research Institute, La Jolla, CA 92037, USA

**Keywords:** *Arenaviridae*, arenavirus, ICTV Report, mammarenavirus, reptarenavirus, taxonomy

## Abstract

*Arenaviridae* is a family for ambisense RNA viruses with genomes of about 10.5 kb that infect mammals, snakes, and fish. The arenavirid genome consists of two or three single-stranded RNA segments and encodes a nucleoprotein (NP), a glycoprotein (GP) and a large (L) protein containing RNA-directed RNA polymerase (RdRP) domains; some arenavirids encode a zinc-binding protein (Z). This is a summary of the International Committee on Taxonomy of Viruses (ICTV) report on the family *Arenaviridae*, which is available at www.ictv.global/report/arenaviridae.

## Virion

**Fig. 1.**
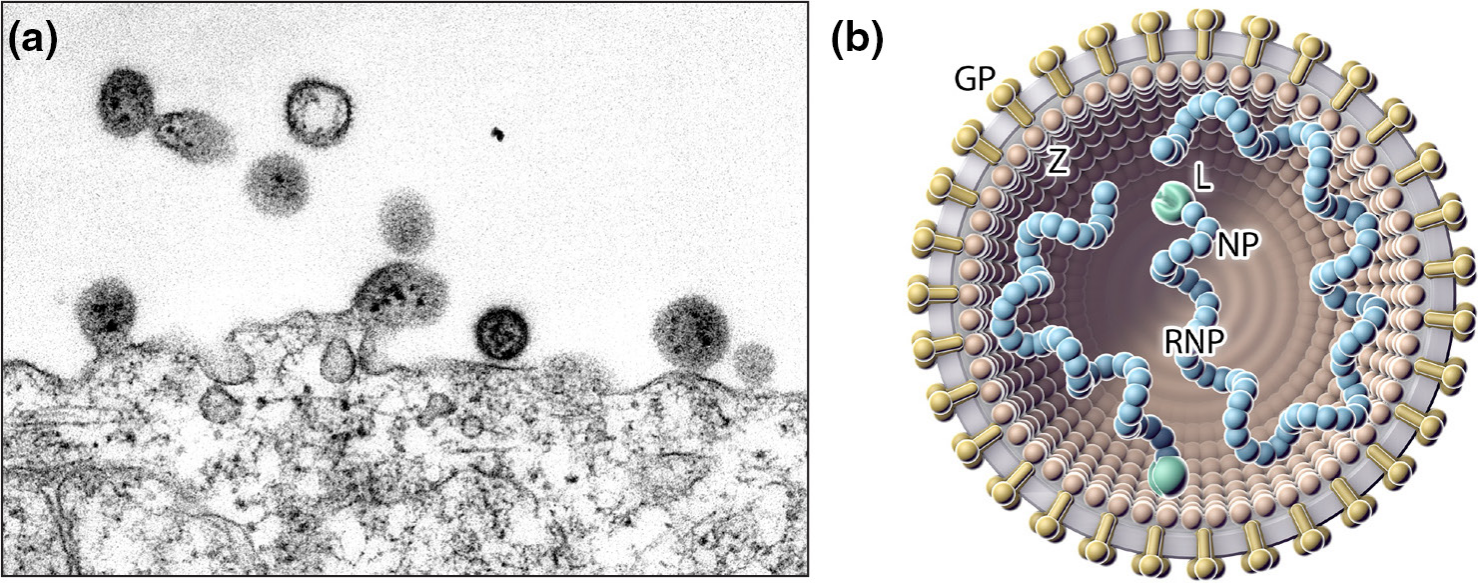
(**a**) Electron micrograph of lymphocytic choriomeningitis virus budding from an infected cell. (**b**) Illustration of a particle in (a) showing the spherical and enveloped particle (grey) that is spiked with glycoproteins (GP, gold) around a layer of zinc-binding proteins (Z, brown). The small (S) and large (L) ribonucleoprotein (RNP) complexes inside the particle consist of nucleoprotein (NP; blue) and large (L; green) protein.

Arenavirids produce virions that are spherical or pleomorphic in shape and 40–200 nm in diameter, with dense lipid envelopes (Table 1 and Fig. 1). The virion surface layer is covered with club-shaped projections that have distinctive stalk and head regions. These projections consist of trimeric spike structures of two virus-encoded membrane glycoprotein (GP) subunits (GP1 and GP2) and, in the case of some arenavirids, a stable signal peptide (SSP). Isolated ribonucleoprotein (RNP) complexes are organized into ‘beads-on-a-string’-like structures [1, 2].

**Table 1.** Characteristics of members of the family *Arenaviridae*

Example	lymphocytic choriomeningitis virus (S: AY847350; L: AY847351), species *Mammarenavirus choriomeningitidis*, genus *Mammarenavirus*
Genome	Two or three single-stranded, usually ambisense, RNA molecules (segments): small (S), medium (M), and large (L)
Replication	Ribonucleoprotein complexes containing anti-genomic RNA serve as templates for synthesis of genomic RNA
Translation	From capped and non-polyadenylated mRNAs. The 5′ cap structure is obtained via cap-snatching from cellular mRNAs
Host range	Fish (antennaviruses), mammals (mammarenaviruses), reptiles (hartmaniviruses and reptarenaviruses), and potentially also ticks
Taxonomy	Realm *Riboviria,* kingdom *Orthornavirae*, phylum *Negarnaviricota*, class *Ellioviricetes*, order *Bunyavirales*; >4 genera and >59 species

## Genome

**Fig. 2.**
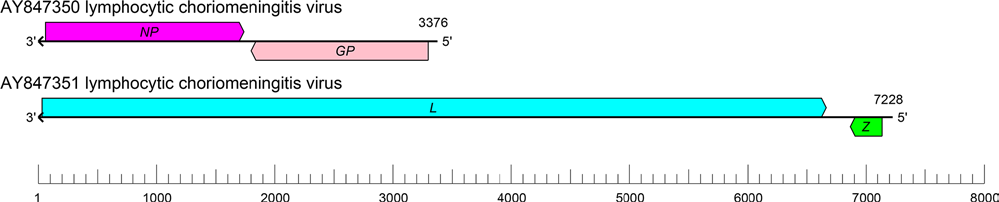
Mammarenavirus genome. The ends of both segments are complementary at their termini, likely promoting the formation of panhandle RNP complexes within the virion. *GP*, glycoprotein gene; *L*, large protein gene; *NP*, nucleoprotein gene; *Z*, zinc-binding protein gene.

Arenavirid genomes consist of two or three single-stranded, typically ambisense, RNA segments (small [S], medium [M], and large [L]). Some of these RNAs encode two proteins in non-overlapping open reading frames of opposite polarities that are separated by non-coding intergenic regions (IGRs) (Fig. 2). The S RNA encodes a nucleoprotein (NP) in the virus genome-complementary strand and, in many cases, a virus glycoprotein precursor (GPC) in the virus genome-sense strand. The L RNA segment encodes a large (L) protein in the virus genome-complementary strand and, in some cases, a zinc-binding protein (Z) in the virus genome-sense sequence [1–3].

## Replication

Arenavirions attach to cell-surface receptors or attachment factors and enter via the endosomal route. Some viruses engage intracellular receptors in endosomes. pH-dependent fusion with late endosomes releases the virion RNP complex into the cytoplasm. The virus RNP directs both RNA genome replication and gene transcription. During replication, L protein reads through the IGR transcription–termination signal and generates uncapped antigenomic and genomic RNAs. Transcription of mRNAs encoding GPC and Z occurs only after the first round of virus replication, during which S and L antigenomes are produced. Arenavirid mRNAs lack 3′-terminal poly(A) tracts but have several non-templated 5′ bases, consistent with the use of a cap-snatching mechanism to initiate transcription. Virion budding occurs from the cellular plasma membrane, thereby providing the virion envelope [1, 2].

## Taxonomy

Current taxonomy: ictv.global/taxonomy. The family *Arenaviridae* is included in the negarnaviricot order *Bunyavirales*. Arenavirids are most closely related to mypovirids, nairovirids, phenuivirids and wupedevirids. Arenavirids differ from most other bunyavirals by having segmented genomes with ambisense organization. The family includes several genera and >59 species. Some arenavirids can cause severe diseases in humans (e.g. Lassa fever) [4]. Other arenavirids cause disease in captive snakes [3, 5].

## Resources

Full ICTV Report on the family *Arenaviridae*: www.ictv.global/report/arenaviridae.
